# Gait Stability Training in a Virtual Environment Improves Gait and Dynamic Balance Capacity in Incomplete Spinal Cord Injury Patients

**DOI:** 10.3389/fneur.2018.00963

**Published:** 2018-11-20

**Authors:** Rosanne B. van Dijsseldonk, Lysanne A. F. de Jong, Brenda E. Groen, Marije Vos-van der Hulst, Alexander C. H. Geurts, Noel L. W. Keijsers

**Affiliations:** ^1^Department of Research, Sint Maartenskliniek, Nijmegen, Netherlands; ^2^Department of Rehabilitation, Cognition and Behavior, Radboud University Medical Center, Donders Institute for Brain, Nijmegen, Netherlands; ^3^Department of Rehabilitation, Sint Maartenskliniek, Nijmegen, Netherlands

**Keywords:** spinal cord injury, virtual reality, gait, balance, stability, ambulatory, rehabilitation, walking

## Abstract

Many patients with incomplete spinal cord injury (iSCI) have impaired gait and balance capacity, which may impact daily functioning. Reduced walking speed and impaired gait stability are considered important underlying factors for reduced daily functioning. With conventional therapy, patients are limited in training gait stability, but this can be trained on a treadmill in a virtual environment, such as with the Gait Real-time Analysis Interactive Lab (GRAIL). Our objective was to evaluate the effect of 6-weeks GRAIL-training on gait and dynamic balance in ambulatory iSCI patients. In addition, the long-term effect was assessed. Fifteen patients with chronic iSCI participated. The GRAIL training consisted of 12 one-hour training sessions during a 6-week period. Patients performed 2 minute walking tests on the GRAIL in a self-paced mode at the 2nd, and 3rd (baseline measurements) and at the 12th training session. Ten patients performed an additional measurement after 6 months. The primary outcome was walking speed. Secondary outcomes were stride length, stride frequency, step width, and balance confidence. In addition, biomechanical gait stability measures based on the position of the center of mass (CoM) or the extrapolated center of mass (XCoM) relative to the center of pressure (CoP) or the base of support (BoS) were derived: dynamic stability margin (DSM), XCoM-CoP distance in anterior-posterior (AP) and medial-lateral (ML) directions, and CoM-CoP inclination angles in AP and ML directions. The effect of GRAIL-training was tested with a one-way repeated measures ANOVA (α = 0.05) and *post-hoc* paired samples *t*-tests (α = 0.017). Walking speed was higher after GRAIL training (1.04 m/s) compared to both baseline measurements (0.85 and 0.93 m/s) (*p* < 0.001). Significant improvements were also found for stride length (*p* < 0.001) and stability measures in AP direction (XCoM-CoP_AP_ (*p* < 0.001) and CoM-CoP_AP−angle_ (*p* < 0.001)). Stride frequency (*p* = 0.27), step width (*p* = 0.19), and stability measures DSM (*p* = 0.06), XCoM-CoP_ML_ (*p* = 0.97), and CoM-CoP_ML−angle_ (*p* = 0.69) did not improve. Balance confidence was increased after GRAIL training (*p* = 0.001). The effects were remained at 6 months. Increased walking speed, stride length, AP gait stability, and balance confidence suggest that GRAIL-training improves gait and dynamic balance in patients with chronic iSCI. In contrast, stability measures in ML direction did not respond to GRAIL-training.

## Introduction

Approximately 60% of the patients with a spinal cord injury (SCI) suffer an incomplete lesion (1). In the chronic phase of an incomplete SCI (iSCI) many patients will encounter deficits at and below the level of the lesion such as muscle weakness, spasticity, and impaired muscle coordination (2). These deficits can impact on functional ambulation (3) and social participation (4). For functional ambulation, walking speed is considered one of the most important parameters (5). Generally, iSCI patients walk at a low preferred walking speed (5) and with a deviant walking pattern (2, 6). One of the underlying causes of the reduced walking performance is impaired balance (7, 8). The high incidence of falls, ranging from 39 to 75% (9, 10), supports the impaired balance in ambulatory patients with iSCI.

Frequently, an important goal of rehabilitation is to improve balance and walking speed. Various interventions and training approaches aiming to improve walking performance in iSCI patients have been introduced and all approaches show some improvement without supremacy of one intervention over others (11). Typical examples of balance and walking training are individual physical therapy and (body-weight-supported) treadmill training. However, these therapies are limited in training patients to react to environmental circumstances without challenging their balance capacity to individual limits.

In recent years, training in a virtual environment has been introduced in rehabilitation (12, 13). In these virtual environments, a simulation of challenging real life situations (such as walking in a forest) can be presented without exposing the user to the direct danger of falling. In this way, patients are given the opportunity to train their gait and balance capacities by exploring their boundaries in a challenging and safe environment (14). Training in virtual environments will provide patients with important prerequisites for motor rehabilitation, such as repetitive practice, feedback about performance, and motivation to endure practice (12). In the virtual environment tasks involving precision stepping, obstacle avoidance, and/or reacting to perturbations, often referred to as “gait adaptability training,” can be performed in quick succession. Such training is highly relevant to relearn daily activities such as walking on uneven surfaces or in crowded places, where people need to adapt their walking speed and walking pattern to environmental circumstances (15–18). Previous research shows that gait adaptability training can improve functional ambulation by preventing falls (19) and improving gait stability in elderly, stroke patients, and patients with Parkinson's disease (20–23). In iSCI patients training precision stepping has been shown to improve walking capacity measured with the SCI functional ambulation profile [SCI-FAP, (24)]. More recently, however, Fox and colleagues concluded that the efficacy of gait adaptability training on walking and balance function should be further investigated (25).

Although walking speed is considered to be the most important characteristic of walking performance, balance capacity is a key element of functional ambulation as well (7, 8). Studies that focused on balance in iSCI patients often used the Berg Balance Scale as a primary outcome (8, 26, 27). The Berg Balance Scale is easy to use, but it is known to have a ceiling effect (28) and assesses balance in rather static situations. Measuring gait stability is more complex and often requires additional equipment such as force plates and a motion capture system. Some virtual reality gait training devices, such as the GRAIL (Gait Real-time Analysis Interactive Lab), can be used as both a training and measurement device. The GRAIL consists of an instrumented dual belt treadmill with two embedded force plates and an eight-camera VICON motion capture system (VICON, Oxford, United Kingdom). The self-paced mode of the GRAIL allows patients to vary the treadmill speed during walking, which induces a natural way of walking (29), especially when a visual flow is presented in the virtual environment (30). When reflective markers are adhered to the participants body, the marker data can be captured for objective offline movement analysis. In addition to walking speed, more complex biomechanical measures related to gait stability can be derived. Previous studies showed that these biomechanical gait stability measures were able to distinguish between elderly with and without balance problems (31, 32), between above-knee amputees and control subjects (33), and between more and less affected stroke patients (34). However, the responsiveness of the stability measures to gait training is unknown.

The main objective of this study was to evaluate the effect of 6 weeks GRAIL training on gait and dynamic balance capacity in ambulatory patients with chronic iSCI. In addition, the long-term effect was assessed 6 months after GRAIL training. Walking speed was used as a primary outcome, while other spatio-temporal parameters and gait stability measures were assessed as secondary outcome parameters. We hypothesized that GRAIL training will result in an improved walking speed and gait stability.

## Materials and methods

### Participants

Patients with iSCI who were referred to GRAIL training by a rehabilitation physician in the Sint Maartenskliniek between June 2016 and December 2017 were eligible to participate in this study. Eligible persons were adults in the chronic phase (>6 months) with an iSCI [American Spinal Injury Association Impairment Scale (AIS) C or D] who could walk independently for 2 min without assistance [Functional Ambulation Categories (FAC) ≥3]. Patients were excluded if (i) they were not able to walk in the self-paced mode of the GRAIL without using the handrails, (ii) had other neurological or lower limb impairments in addition to the iSCI, (iii) had vision problems, or (iv) had walking and/or balance problems prior to the iSCI. The exclusion criteria (ii), (iii), and (iv) were checked by the researcher through questions. All participants gave written informed consent in accordance with the Declaration of Helsinki. The study was approved by the regional medical ethics committee of Arnhem-Nijmegen (2016–2474) and by the internal review board of the Sint Maartenskliniek.

### Equipment

**Figure 1:**
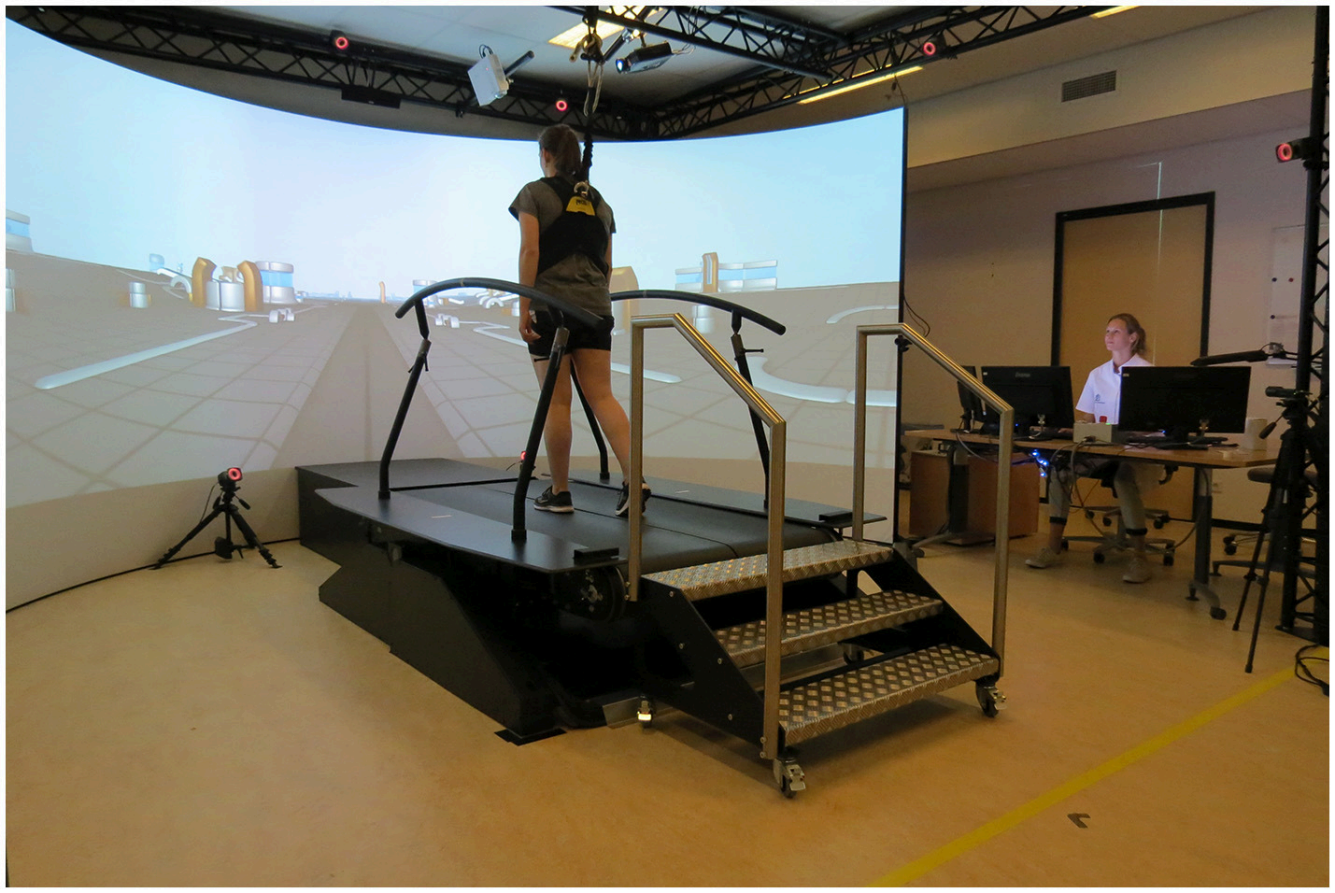
Gait Real-time Analysis Interactive Lab (GRAIL) at the Sint Maartenskliniek. Both persons have given their written and informed consent for publication of the picture.

All training sessions and measurements were performed on the GRAIL at the Sint Maartenskliniek in Nijmegen (Figure 1). The GRAIL consisted of an instrumented dual belt treadmill with two embedded force plates and an eight-camera VICON motion capture system (VICON, Oxford, United Kingdom). The platform was able to move in several directions to generate mechanical perturbations. In front of the treadmill, virtual reality environments were projected on a 180° semi-cylindrical screen. Reflective markers were adhered to the patients to interact with the virtual environment and to capture kinematic data. The GRAIL system was controlled and the visual information was matched to the treadmill speed with the D-flow software (Motek Forcelink, Amsterdam, the Netherlands, version 3.22.1). To assure safety, patients wore a safety harness without body weight support.

### Protocol

#### Intervention

The GRAIL training consisted of 12 1-h training sessions spread over a 6-week period. Per training session one physical therapist guided the training and a maximum of two physical therapists were responsible for all training sessions given to one patient. All physical therapists were certified GRAIL operators. Each training was individualized, as the physical therapist chose the training applications based on the specific rehabilitation goal and current level of the patient. For instance, patients who had problems maintaining their balance in stance typically performed applications in which they had to shift their weight, whereas patients with gait adaptability problems performed applications in which they had to perform precision stepping or obstacle avoidance. During the GRAIL training multiple applications were performed and after each training session the physical therapist documented the type and level of the performed applications. The applications were categorized in three themes; “gait adaptability,” “walking^+^,” “balance in stance” (see Table 2). The first GRAIL training session was used for familiarization with the GRAIL system. From the second to the last training session, training intensity, and complexity were gradually and individually increased.

#### Gait measurements

**Figure 2:**
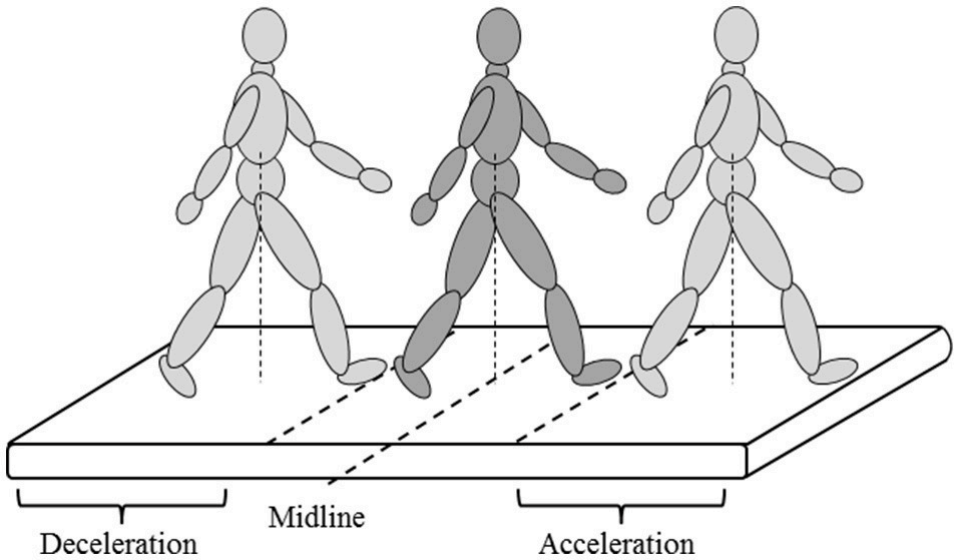
Self-paced mode on the GRAIL.

To evaluate the effect of GRAIL training on gait and balance, patients performed the 2 minute walking test (2MWT) at the 2nd, 3rd, and last (12th) training sessions (baseline 1, baseline 2, and post measurement, respectively). For familiarization with the task, the 2nd baseline measurement (at the 3rd training session) was added to neutralize early learning (or task adaptation) effects. To evaluate the long-term effect of GRAIL training on gait and balance, patients performed one additional 2MWT on the GRAIL 6 months after the last training session (follow-up). Patients performed the 2MWT in the self-paced mode on the GRAIL, which allowed them to walk at a self-selected speed. In the self-paced mode, the speed of the treadmill was automatically controlled using the anterior-posterior (AP) position of the pelvis markers and the AP midline of the treadmill. Walking forward or backward relative to the midline resulted in an acceleration or deceleration of the treadmill, respectively (Figure 2). Before measuring the 2MWT, patients received some explanation about the self-paced mode and performed a few practice trials to reach their preferred walking speed in a similar manner as in the study of Plotnik and colleagues (30). During the 2MWT on the GRAIL, patients were instructed to walk as far as possible at a comfortable walking speed in 2 min. Patients received no feedback about their walking speed.

**Figure 3:**
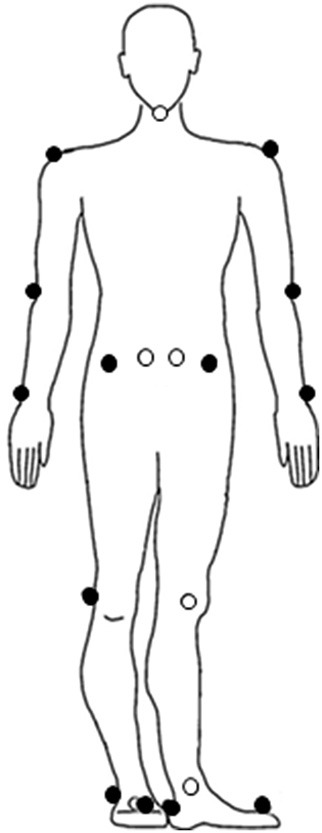
Placement of the reflective markers.

The self-paced mode was set at the lowest sensitivity value of 1.0 (setting ranged between 1 and 5). The maximum acceleration and deceleration of the treadmill was set at 0.25 m/s^2^. Before the start of the 2MWT, 19 reflective markers were adhered to the following anatomical landmarks: left and right acromion process, humeral lateral epicondyle, ulnar styloid process, anterior superior iliac spine (ASIS), posterior superior iliac spine (PSIS), femoral lateral epicondyle, lateral malleolus, metatarsal II, calcaneus, and 7th cervical vertebra (Figure 3). The data of the reflective markers was sampled at a frequency of 100 Hz and the sample frequency of the force plates was 1,000 Hz. The data of the reflective markers were labeled using VICON Nexus 2.4 and analyzed using MATLAB R2017b. A zero lag second-order Butterworth filter with a cut-off frequency of 10 Hz was used to filter the marker data. A cut-off frequency of 7 Hz was used for the force data. Before the patients walked at their preferred walking speed, the treadmill had to accelerate. To remove the acceleration phase, the first 20 s of the 2MWT were removed before data analysis.

##### Spatiotemporal parameters

The primary outcome was walking speed defined as the average treadmill speed (m/s). Other spatiotemporal gait parameters (stride length, step width, and stride frequency) were used as secondary outcome parameters. Stride length (cm) was determined as the average AP-distance between the heel markers at two consecutive heel strikes on the same side. Step width (cm) was determined as the average ML-distance between the heel markers at heel strike. Stride frequency (strides/s) was defined as the inverse of the interval between heel strikes of the same foot. Heel strikes were defined as the instant that the calcaneus marker started moving backwards.

##### Gait stability measures

The stability measures used in the current study were based on the position of the center of mass (CoM) or the extrapolated center of mass (XCoM) relative to the center of pressure (CoP) or the base of support (BoS), during a specific moment of the gait cycle (e.g., double support or heel strike). The position of the CoM depends on sex, body posture, and direction of the limbs. In this study the CoM was calculated using 19 reflective markers according to a method first described by Tisserand et al. (35). The XCoM takes the position and velocity of the CoM into account and is used to formulate requirements for gait stability (36). To calculate the XCoM, the equation of Hof et al was used (36, 37):

$$\begin{array}{l} XCoM\;=\;CoM^{\prime }\;+\;\frac{vCoM}{\sqrt{\frac{g}{l}}} \end{array}$$

*CoM'* represents the ground projection of the CoM, *vCoM* the velocity of the CoM, *g* = 9.81 m/s^2^, and *l* the maximum height of the CoM (36, 37). The CoP is the centroid of pressure distribution on the plantar surface of the foot and has been used to identify balance control during posture and gait (38). The equation of Sloot et al was used to calculate the CoP for both force plates (39):

$$\begin{array}{lll} CoP_{AP} & = & \;\frac{F_{AP}*CoP_{V}-\;M_{ML}}{F_{V}}\;\&\\ {CoP}_{ML} & = & \;\frac{F_{ML}*CoP_{V}-\;M_{AP}}{F_{V}} \end{array}$$

*F* represents the force in anterior-posterior (_*AP*_), medial-lateral (_*ML*_), and vertical (_*V*_) directions, *M* the moment of force, and *CoP*_*V*_ the vertical distance between the surface of the treadmill belt and the force plates (39). During the double support phase, the weighted average of the CoP in ML and AP directions was calculated based on the CoP of both force plates.

**Figure 4:**
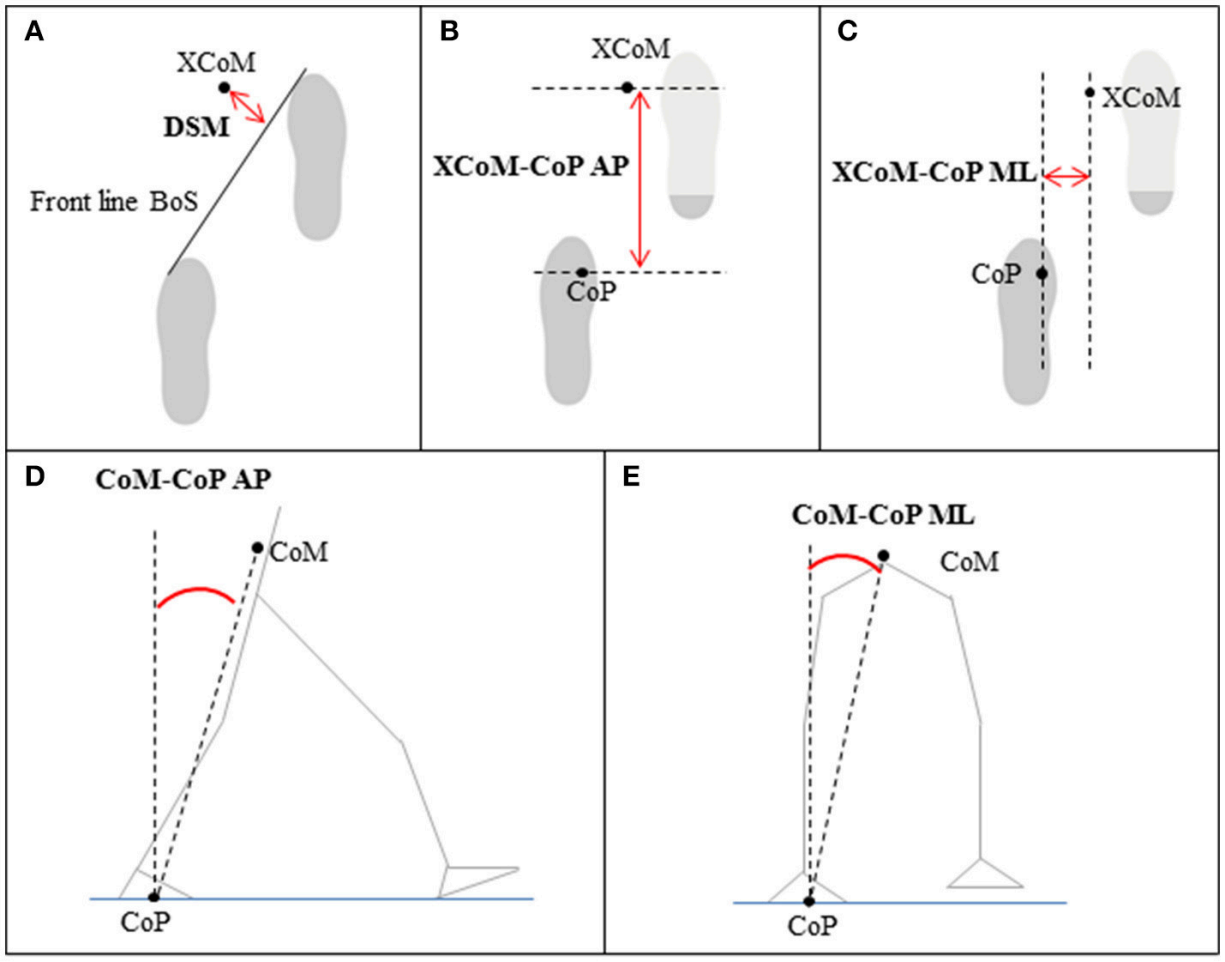
Gait stability measures based on the position of the center of mass (CoM), extrapolated center of mass (XCoM), center of pressure (CoP), and/or base of support (BoS) relative to each other; **(A)** dynamic stability margin (DSM), **(B,C)** XCoM-CoP distance in anterior-posterior (AP) and medial-lateral (ML) direction, **(D,E)** CoM-CoP inclination angles in AP and ML direction.

In this study the following five gait stability measures were calculated: the dynamic margin of stability (DSM) (34), the XCoM-CoP distance in AP and ML directions (32, 33), and the CoM-CoP inclination angles in AP and ML directions (31). In general, better gait stability is characterized by a position of the XCoM far in front of the BoS in the AP direction, which suggests that patients are confident in walking at higher speeds with longer steps. In the ML direction, better gait stability is characterized by a position of the XCoM closer to the boundaries of the BoS, often accompanied by walking with a smaller step width. The DSM was calculated as the average of the shortest distance between the front line of the BoS (i.e., the line between the two metatarsal II markers) and the XCoM during double support (34) (See Figure 4A for a visual representation). A large positive DSM represents better balance control than a negative DSM (i.e., XCoM within the BoS) or smaller DSM. The distances between the XCoM and CoP were calculated at each heel strike in AP (Figure 4B) and ML directions (Figure 4C) separately (32, 33). A larger XCoM-CoP_AP_ distance and a smaller XCoM-CoP_ML_ distance reflect better balance control. The CoM-CoP inclination angles were calculated from the angle between the position of the CoM and the vertical line through the CoP (31). The peak inclination angles were defined as the range between the maximum and minimum inclination angles in AP (Figure 4D) and ML directions (Figure 4E) of each gait cycle. A larger peak inclination angle in the AP direction and a smaller peak inclination angle in the ML direction represent better balance control.

#### Balance confidence assessment

Balance confidence was assessed with the activities specific balance confidence (ABC) (0–100) scale (40, 41) at the second training session (before the first baseline measurement), at the last training session (after the post measurement) and at 6 months after the last training session (after the follow-up measurement).

### Statistical analysis

#### Effect of GRAIL training

The spatiotemporal gait parameters (walking speed, stride length, step width, and stride frequency) and gait stability measures (DSM, XCoM-CoP_AP_ distance, XCoM-CoP_ML_ distance, CoM-CoP_AP−angle_, and CoM-CoP_ML−angle_) were analyzed using descriptive statistics (mean and standard deviation). Differences in the spatiotemporal gait parameters and gait stability measures between the three measurements (baseline 1, baseline 2, and post measurement) were assessed with a one-way (factor Time) repeated measures ANOVA (α = 0.05). If the assumption of sphericity was violated, the degrees of freedom were corrected using Greenhouse-Geisser correction and the Pillai's Trace value (V) was given. In the case of a significant effect of Time, paired samples *t*-tests with Bonferroni correction (α = 0.017) were performed to determine which measurements were different from each other. In case the assumption of normality was violated, median, and ranges were calculated and a non-parametric Friedman test (α = 0.05) with Wilcoxon signed-rank *post-hoc* test (α = 0.017) was performed. The effect of GRAIL training on balance confidence (the scores on the pre and post ABC-scale) was tested with paired samples *t*-test (α = 0.05). *F* and *t*-values were given when the repeated measures ANOVA and paired samples *t*-test were used, while *X*${}_{F}^{2}$ and *T* were given for the non-parametric Friedman test and Wilcoxon signed-rank test.

#### Long-term effect of GRAIL training

The long-term effect of GRAIL training was evaluated by testing the differences in spatiotemporal gait parameters (walking speed, stride length, step width, and stride frequency) and gait stability measures (DSM, XCoM-CoP_AP_ distance, XCoM-CoP_ML_ distance, CoM-CoP_AP−angle_, and CoM-CoP_ML−angle_) between the post and follow-up measurements as well as between the baseline 2 and follow-up measurements using paired samples *t*-tests (α = 0.05). When the assumption of normality was violated, median, and ranges were calculated and a non-parametric Wilcoxon signed-rank test (α = 0.05) was performed. *t*-values were given when the paired samples *t*-test was used, while *T* was given for the non-parametric equivalent, the Wilcoxon signed-rank test.

## Results

### Participants

**Figure 5:**
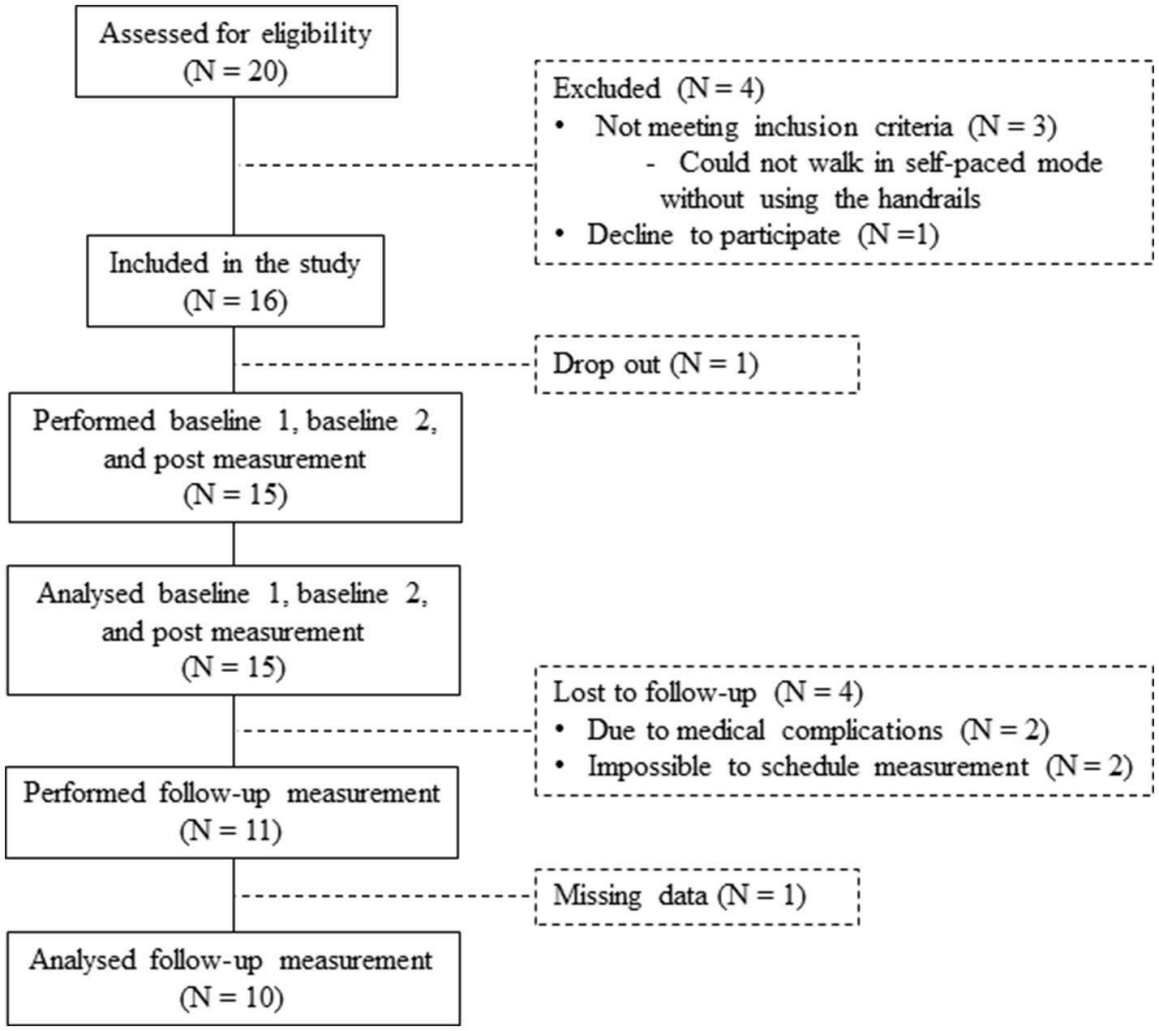
Flow diagram of patients in the study.

In total 20 patients were assessed for eligibility in the study. Three patients were ineligible because they could not walk in the self-paced mode without using the handrails (exclusion criteria). One patient declined to participate. Sixteen patients were included in the study. One dropped out before completing the post measurement, resulting in 15 patients who performed the baseline 1, baseline 2, and post measurements (Figure 5). An overview of the patient characteristics is given in Table 1.

**Table 1:** Patient characteristwics.

	**Performed baseline 1, baseline 2 and post measurement (*N* = 15)**	**Completed follow-up (*N* = 10)**
Sex (male/female)	11/4	9/1
Age (years), mean (SD)	59 (12)	59 (12)
Post-injury (months), mean (SD)	42 (48)	42 (46)
AIS^*^(C/D)	2/13	1/9
BMI^**^, mean (SD)	27 (2)	26 (2)
FAC^***^(3/4/5)	1/6/8	1/4/5
More affected side (left/ right/ no difference)	7/3/5	5/1/4

* *AIS, American Spinal Injury Association Impairment Scale*.

** *BMI, Body-Mass Index*.

*** *FAC, Functional Ambulatory Category*.

### Content of GRAIL training

Two patients received 9 and 8 GRAIL training sessions instead of the scheduled 12 training sessions. These patients canceled some training sessions at short notice, which made it impossible to reschedule the sessions within the training period. The other 13 patients received 12 GRAIL training sessions. In total 30 different applications were performed during the GRAIL training. The themes of these applications were categorized in “gait adaptability” (13 applications), “walking^+^” (8 applications), and “balance in stance” (9 applications). On average, patients performed 3.7 ± 0.9 applications during one training session, of which 1.4 ± 0.4 were gait adaptability applications, 1.3 ± 0.4 walking^+^ applications, and 1.0 ± 0.5 balance in stance applications. The most frequently practiced applications for gait adaptability were “Microbes” (42%), for walking^+^ “Perturbations” (23%), and for balance in stance “Traffic jam” (42%). An explanation of the most frequently practiced applications per theme is depicted in Table 2.

**Table 2:** Most frequently performed applications on the GRAIL per theme.

**Theme**	**Gait adaptability**	**Walking^+^**	**Balance in stance**
Application	“Microbes”	“Perturbations”	“Traffic jam”
Virtual environment	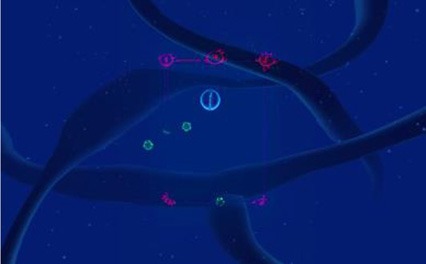	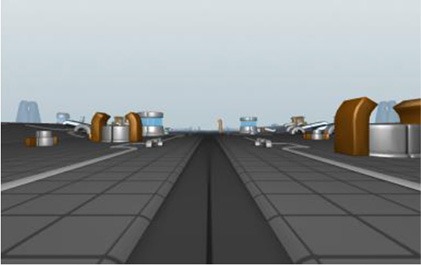	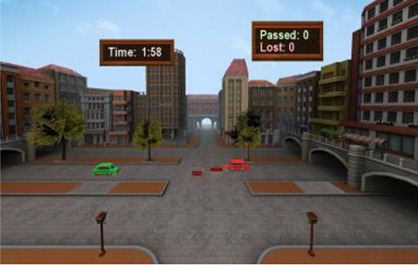
Task	Collecting as many green microbes by changing one's position on the treadmill during gait.	Walking on the treadmill and responding as quickly and accurately as possible to the perturbations.	Letting cars cross the road by lifting the feet in stance.
Training purpose	Accelerate and decelerate, change walking direction, adapt step length, avoid obstacles, and perform foot clearance.	React to: sideward translation of the treadmill, treadmill pitch forward or backward, acceleration or deceleration of one treadmill belt.	Shift weight, perform foot clearance, and initiate steps.

### Effect of GRAIL training

#### Spatiotemporal parameters

**Figure 6:**
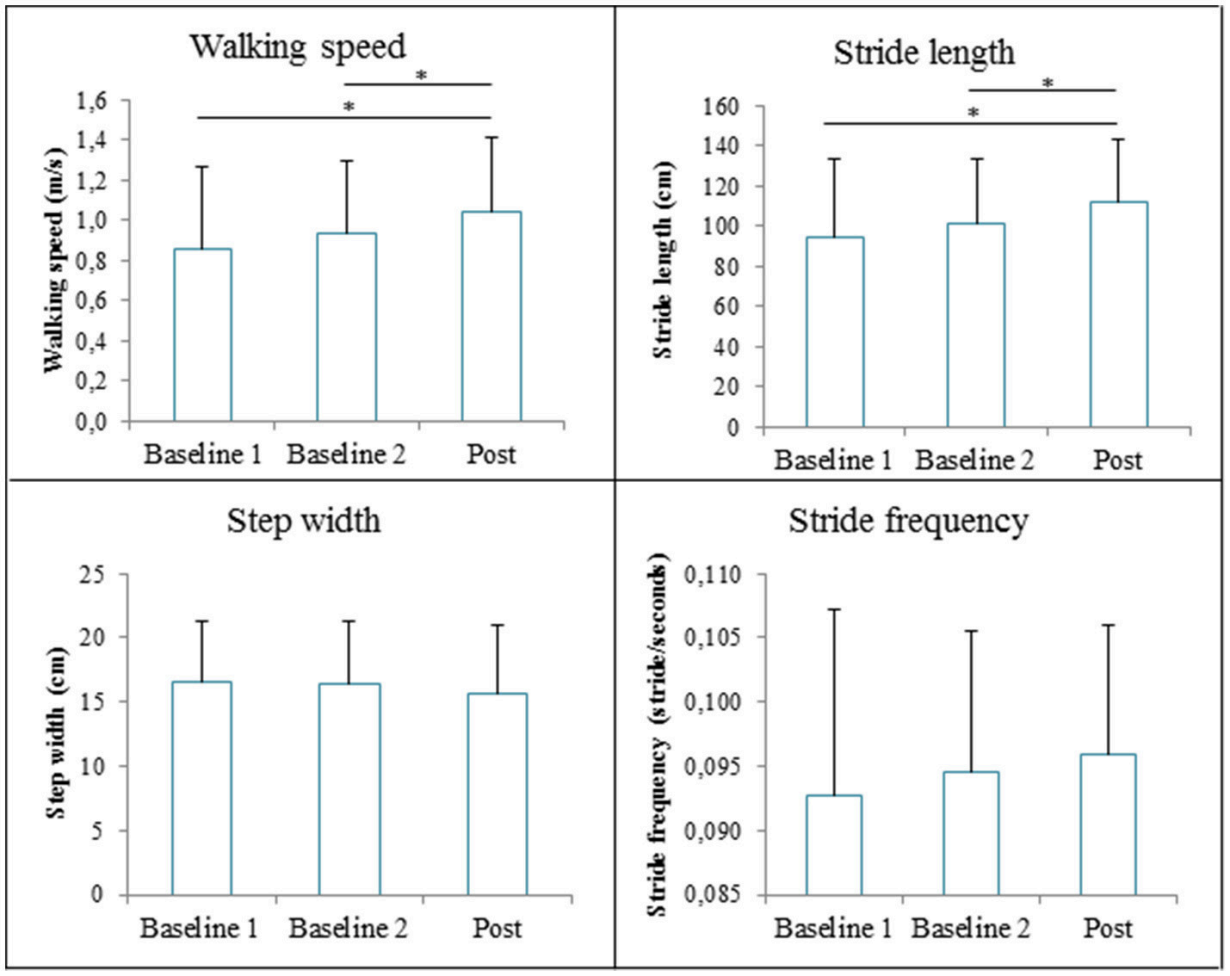
The spatiotemporal gait parameters (means and standard deviations) during the 6 weeks GRAIL training. *Asterisk indicates a *post-hoc* significant difference (α = 0.017).

The repeated measures ANOVA revealed significant Time effects of GRAIL training on walking speed [*F*_(2, 28)_ = 18.53, *p* < 0.001]. *Post-hoc* analysis showed that the mean walking speed was significantly higher at post measurement (1.04 ± 0.38 m/s) compared to baseline 1 (0.85 ± 0.41 m/s, *p*<*0.001*) and baseline 2 (0.93 ± 0.37 m/s, *p* = 0*.003*). There was a significant effect of GRAIL training on the stride length, [*F*_(2, 28)_ = 15.76, *p*<*0*.001]. Stride length was significantly larger at the post measurement (112 ± 31cm) compared to baseline 1 (94 ± 39 cm, *p*<*0.001*) and baseline 2 (101 ± 33 cm*, p* = 0.002). Stride frequency [*V* = 0.18, *F*_(2, 13)_ = 1.45, *p* = 0.27] and step width [*F*_(2, 28)_ = 1.76, *p* = 0.19] were not significantly affected by GRAIL training. The spatiotemporal gait parameters at the three measurements are shown in Figure 6.

#### Gait stability measures

**Figure 7:**
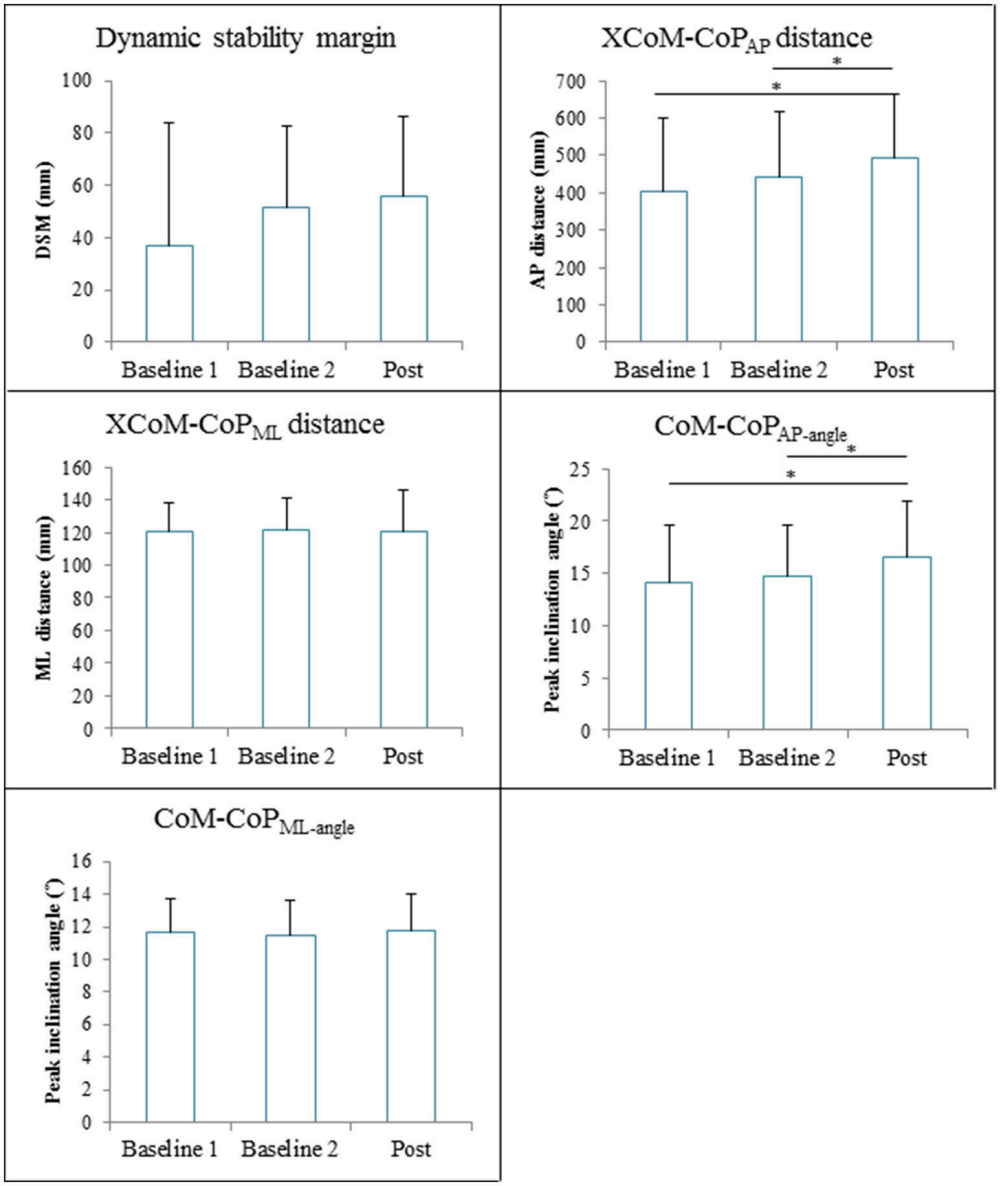
Gait stability (means and standard deviations) during 6 weeks GRAIL training. A visual representation of the gait stability measures is given in Figure 4. ^*^Asterisk indicates a *post-hoc* significant difference (α = 0.017).

The repeated measures ANOVA revealed significant Time effects on XCoM-CoP_AP_ distance [*F*_(2, 28)_ = 19.48, *p* < 0.001] and CoM-CoP_AP−angle_ [*F*_(2, 28)_ = 15.90, *p* < 0.001]. The XCoM-CoP_AP_ distance was significantly higher at post measurement (491 ± 175 mm) compared to baseline 1 (404 ± 195 mm, *p*<*0.001*) and baseline 2 (441 ± 177 mm, *p* = 0.003). The CoM-CoP_AP−angle_ was significantly higher at post measurement (16.6 ± 5.3°) compared to baseline 1 (14.1 ± 5.5°, *p*<*0.001*) and baseline 2 (14.7 ± 4.8°, *p* = 0.003). The Time effect on the DSM nearly reached significance [*V* = 0.36, *F*_(2, 13)_ = 3.64, *p* = 0.06], whereas the XCoM-CoP_ML_ distance [*F*_(2, 28)_ = 0.003, *p* = 0.97] and CoM-CoP_ML−angle_ [*F*_(2, 28)_ = 0.38, *p* = 0.69] were not significantly affected by GRAIL training. Figure 7 gives an overview of the gait stability measures across time.

#### Balance confidence

**Figure 8:**
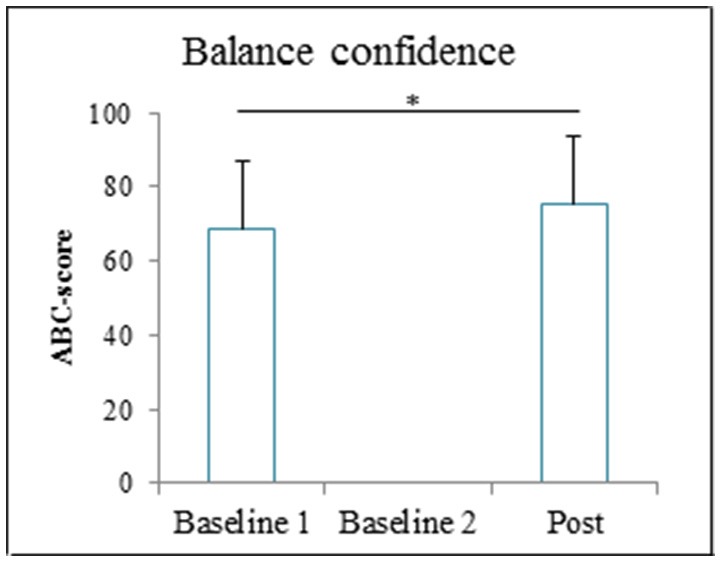
Activities specific balance confidence (ABC) score (means and standard deviations) before and after the 6-weeks GRAIL training. ^*^Asterisk indicates a significant difference (α = 0.05).

Patients' balance confidence significantly increased after GRAIL training (76 ± 18), compared to baseline (69 ± 18) [*t*_(13)_ = −4.55, *p* = 0.001]. The balance confidence scores before and after GRAIL training are shown in Figure 8.

### Long-term effect of GRAIL training

The follow-up measurement was performed by 11 of the 15 patients (Figure 5). Two patients did not perform the follow-up measurement due to medical complications, which were not related to the GRAIL training. Two other patients were lost to follow-up, because it was impossible to schedule their measurements. As a result of a technical error during the follow-up measurement, the data of one additional patient was missing. Therefore, the results of 10 patients were used for the analysis of the long-term effect of GRAIL training (see Table 1 for patient characteristics).

There was no significant difference in walking speed between post (median 1.13 m/s) and follow-up (median 1.30 m/s) measurement [*T* = 20.50, *p* = 0.48], nor was there a significant difference in stride length [*T* = 27, *p* = 0.96], step width [*t*_(9)_ = 0.82, *p* = 0.43], or stride frequency [*t*_(9)_ = −1.04, *p* = 0.33] between the post measurement and the follow-up measurement. The CoM-CoP_ML−angle_ was significantly smaller in the follow-up (10.3 ± 1.6°) compared to the post measurement (11.4 ± 2.2°), [*t*_(9)_ = 2.4, *p* = 0.04]. The other gait stability measures (DSM, CoM-CoP_AP−angle_ and XCoM-CoP_AP_ and XCoM-CoP_ML_ distances) and balance confidence score were not significantly different at the follow-up measurement compared to the post measurement. An overview of the spatiotemporal gait parameters, gait stability measures and balance confidence are shown in Table 3.

**Table 3:** The spatiotemporal gait parameters, gait stability measures and balance confidence in the baseline 2, post and follow-up measurement (*N* = 10).

	**Baseline 2 measurement (mean ±SD, median [min–max])**	**Post measurement (mean ±SD, median [min–max])**	**Follow-up measurement (mean ±SD, median [min–max])**	***p* baseline 2 - follow-up**	***p* post - follow-up**
Walking speed (m/s)	0.89 [0.36–1.45]	1.13 [0.44–1.53]	1.30 [0.34–1.48]	0.03^*^	0.48
Stride length (cm)	103 [57–144]	118 [68–145]	128 [51–144]	0.04^*^	0.96
Step width (cm)	14.4 ± 4.7	13.7 ± 5.0	13.0 ± 4.4	0.19	0.43
Stride frequency (stride/s)	0.93 ± 0.13	0.96 ± 0.04	0.97 ± 0.05	0.07	0.33
DSM (mm)	54 ± 36	60 ± 32	57 ± 44	0.70	0.65
XCoM-CoP_AP_ (mm)	431 [161–718]	511 ± 170	525 ± 201 627 [158–696]	0.07	0.62
XCoM-CoP_ML_ (mm)	121 ± 23	118 ± 26	111 ± 22	0.12	0.15
CoM-CoP_AP−angle_ (°)	15.1 ± 4.6	17.4 ± 5.0	17.7 ± 6.0	0.03^*^	0.77
CoM-CoP_ML−angle_ (°)	10.9 ± 2.3	11.4 ± 2.2	10.3 ± 1.6	0.23	0.04^*^
ABC-score	70.3 ± 19.0	77.1 ± 19.3	74.5 ± 20.4	0.13	0.09

* *Asterisk indicates a significant difference (α = 0.05) in the paired samples t-test (mean ± SD) or Wilcoxon signed-rank test (median [min–max])*.

Walking speed (*p* = 0.03), stride length (*p* = 0.04), and CoM-CoP_AP−angle_ (*p* = 0.03) were significantly higher in the follow-up measurement compared to the baseline 2 measurement. The other outcome measures (step width, stride frequency, DSM, CoM-CoP_ML−angle_ and XCoM-CoP_AP_ and XCoM-CoP_ML_ distances and balance confidence) were not significantly different at the follow-up measurement compared to the baseline 2 measurement (Table 3).

## Discussion

The aim of the present study, was to assess the effects of 6 weeks GRAIL training on gait and dynamic balance capacities in chronic iSCI patients. Walking speed was increased after GRAIL training (1.04 m/s) compared to baseline measurements (0.85 and 0.93m/s). Stride length was increased, but stride frequency and step width did not change. In addition, the stability measures in AP direction (XCoM-CoP_AP_ and CoM-CoP_AP−angle_) were improved after GRAIL training, whereas stability measures in ML direction (XCoM-CoP_ML_ and CoM-CoP_ML−angle_) or combining AP and ML directions (DSM) did not change. Patients' confidence in balance was increased after GRAIL training. At the 6 months follow-up measurement, improvements in walking speed, stride length, and the stability measure CoM-CoP_AP−angle_ remained increased compared to baseline.

In patients with iSCI, restoration of ambulation is considered the most important rehabilitation goal (42). Typically interventions in these patients focus on improving locomotion (43). For functional ambulation in daily life, walking speed is considered one of the most important parameters (5). After GRAIL training, walking speed increased by 0.19 m/s compared to baseline 1 and by 0.11 m/s compared to baseline 2. Although previous studies in chronic iSCI patients used more gait training sessions (on average 45; range 24–58) due to a higher training frequency (on average 4 sessions/week; range 3–5), and a longer training duration (on average 12 weeks; range 8–16), these studies showed increases in walking speed ranging from 0.01 to 0.16 m/s (24, 44–48). To our knowledge, only two interventions in chronic iSCI patients resulted in an increase in walking speed in the 0.11 to 0.19 m/s range (44, 47). These interventions consisted of 48 sessions of resistance training combined with aerobic training resulting in an increase of 0.13 m/s (47) and 39 sessions of body-weight-supported treadmill training resulting in an increase of 0.16 m/s (44). Due to the higher number of training session, a larger training effect can be expected. Despite the limited number of GRAIL training sessions in the present study, patients improved their walking speed significantly. This improvement exceeded the reported minimal clinically important difference (MCID) of 0.10 m/s (49) in 10 out of 15 participants, reflecting clinical meaningful effects in these participants. Moreover, the effect on walking speed, stride length, and the stability measure CoM-CoP_AP−angle_ were still present 6 months after the last training session. Therefore, randomized controlled trials (or studies with a randomized cross-over design) are warranted to investigate the intervention effects of GRAIL training compared to other gait training interventions in patients with iSCI.

The increase in walking speed, accompanied by an increase in stride length but with a constant stride frequency, suggests that patients learned to take larger steps because they felt more confident after GRAIL training. Indeed, the statistically significant increase in balance confidence score supports the notion that patients felt more safe after the training. However, only one participants exceeded the reported minimal detectable change (MDC) of 14.87 (50). Therefore, the effect of GRAIL training on balance confidence seems to be relatively small. Nevertheless, the significant increase in balance confidence on a group level could be due to improved gait stability. In the current study, recently developed biomechanical stability measures were used to assess gait stability. In previous studies, these stability measures appeared to be significantly different between more and less impaired stroke patients (34), above-knee amputees and healthy subjects (33), and elderly with and without balance problems (31, 32). To our knowledge, this is the first study to assess these stability measures during gait in a pre- and post-intervention design. The gait stability in AP direction was significantly increased after 6 weeks GRAIL training. Because a high correlation between walking speed and AP gait stability can be expected and because we did not perform a post measurement in which patients walked at baseline speed, it cannot be definitively concluded whether patients walked faster after GRAIL training because of improved gait stability or vice versa. In the present study, the stability measures in the ML direction did not differ between the measurements. Future research should test the clinical value of gait stability measures in different directions in patients with iSCI.

Various factors could be responsible for the improved walking speed after GRAIL training. Firstly, patients performed tasks in a complex virtual environment, in which visual and auditory feedback were provided. Patients could make corrections and enhance their motor performance according to the feedback in real time (based on knowledge of performance) as well as at the end of the application (based on knowledge of results) (12). It is well accepted that feedback improves the rate of motor learning (51). Feedback can be particularly beneficial in patients with iSCI in which internal feedback (such as from the proprioceptive system) is disturbed (52). Secondly, different training environments can be quickly alternated during GRAIL training. According to Hedel and colleagues, in well-recovered iSCI patients, such as in the current study, rehabilitation programs should train adaptive locomotion in different environments (52). Thirdly, GRAIL training can be personalized and the intensity and complexity of the applications can be gradually increased. We assume that the effects found in the current study are partly due to the personalization of the GRAIL training to the patients' individual goals. Therefore, it does not seen appropriate to standardize the GRAIL training for each patient.

Observed improvements could have been caused by a familiarization effect when walking on the GRAIL in the self-paced mode. To neutralize this effect, two baseline measurements were performed, one at the 2nd and the other at the 3rd GRAIL training sessions. Furthermore, a familiarization protocol with self-paced walking on the GRAIL, similar as in the study of Plotnik and colleagues (30), was performed before the first baseline measurement. In the study of Plotnik and colleagues, healthy participants reached their steady walking speed already after ~24 m when visual flow was presented and reached a walking speed comparable with overground walking after merely 7.5 to 17.5 m (30). In the current study, an increase of 0.08 m/s was seen between the baseline 1 and 2 measurements. This increase could be partly due to familiarization with self-paced walking on the GRAIL. Future research should investigate if familiarization with self-paced walking takes more time in patients with impaired gait stability than in healthy subjects. Important to note is that the self-paced walking was not practiced in the subsequent GRAIL training sessions. Nevertheless, patients further increased their walking speed significantly at the post-measurement compared to baseline 2 by 0.11 m/s. Moreover, at 6 months follow-up, this beneficial effect was still present, suggesting a true effect of GRAIL training.

A limitation of the current study is that the follow-up measurement was completed by only 10 patients and that we did not control co-interventions during the period after the GRAIL training. Another limitation is that we do not know how the effect of GRAIL training has affected gait and dynamic balance capacities during overground walking. Although previous studies concluded that self-paced treadmill walking induces natural gait (29) and that gait speed on a treadmill is comparable to overground walking (30), future research should investigate whether the effect of GRAIL training also extends to overground walking, walking in daily life, and to social participation in ambulatory iSCI patients.

## Conclusion

The increased walking speed, stride length, AP gait stability, and balance confidence suggest that GRAIL training improves gait and dynamic balance capacity in patients with chronic iSCI.

## Author contributions

NK, BG, MV, and AG contributed conception and design of the study. MV was responsible for the patient recruitment. LdJ, RvD, and BG performed the measurements. LdJ organized the database. RvD and NK processed the experimental data, performed the analysis, drafted the manuscript, and designed the figures. NK and BG supervised the project. All authors discussed the results and commented on the manuscript.

### Conflict of interest statement

The authors declare that the research was conducted in the absence of any commercial or financial relationships that could be construed as a potential conflict of interest.
